# Placental weight and birth weight to placental weight ratio in monochorionic and dichorionic growth-restricted and non-growth-restricted twins

**DOI:** 10.6061/clinics/2017(05)02

**Published:** 2017-05

**Authors:** Mariângela Alves Souza, Maria de Lourdes Brizot, Sckarlet Ernandes Biancolin, Regina Schultz, Mário Henrique Burlacchini de Carvalho, Rossana Pulcineli Vieira Francisco, Marcelo Zugaib

**Affiliations:** I Departamento de Ginecologia e Obstetricia, Faculdade de Medicina, Universidade de Sao Paulo, Sao Paulo, SP, BR; II Departamento de Patologia, Hospital das Clinicas HCFMUSP, Faculdade de Medicina, Universidade de Sao Paulo, São Paulo, SP, BR

**Keywords:** Twin Pregnancy, Placental Pathology, Placental Weight, Birth Weight/Placental Weight Ratio, Monochorionic Twin, Growth Restriction

## Abstract

**OBJECTIVE:**

The aim of the present study was to compare the placental weight and birth weight/placental weight ratio for intrauterine growth-restricted and non-intrauterine growth-restricted monochorionic and dichorionic twins.

**METHODS:**

This was a retrospective analysis of placentas from twin pregnancies. Placental weight and the birth weight/placental weight ratio were compared in intrauterine growth-restricted and non-intrauterine growth-restricted monochorionic and dichorionic twins. The association between cord insertion type and placental lesions in intrauterine growth-restricted and non-intrauterine growth-restricted monochorionic and dichorionic twins was also investigated.

**RESULTS:**

A total of 105 monochorionic (intrauterine growth restriction=40; non-intrauterine growth restriction=65) and 219 dichorionic (intrauterine growth restriction=57; non-intrauterine growth restriction=162) placentas were analyzed. A significantly lower placental weight was observed in intrauterine growth-restricted monochorionic (*p*=0.022) and dichorionic (*p*<0.001) twins compared to non-intrauterine growth-restricted twins. There was no difference in the birth weight/placental weight ratio between the intrauterine growth restriction and non-intrauterine growth restriction groups for either monochorionic (*p*=0.36) or dichorionic (*p*=0.68) twins. Placental weight and the birth weight/placental weight ratio were not associated with cord insertion type or with placental lesions.

**CONCLUSION:**

Low placental weight, and consequently reduced functional mass, appears to be involved in fetal growth restriction in monochorionic and dichorionic twins. The mechanism by which low placental weight influences the birth weight/placental weight ratio in intrauterine growth-restricted monochorionic and dichorionic twins needs to be determined in larger prospective studies.

## INTRODUCTION

The placenta is a specialized organ of pregnancy that supports fetal growth and development. Placental disorders are associated with maternal-fetal complications, such as hypertension, diabetes, malformations, fetal anemia or hydrops, congenital infection and fetal growth restriction.

Recent publications have indicated the importance of reference values for singleton and twin placental weight and of the relationship between placental weight and fetal growth 1-3. Similar to singletons, there is a positive relation between birth weight and placental weight for twins 4. Furthermore, compared to singletons, twins have lower birth weights and smaller placentas 2,5. Few studies have distinguished placental weight in dichorionic (DC) and monochorionic (MC) twin pregnancies 1,3,6, and they have reported conflicting results; Pinar et al. did not observe a difference in placental weight between MC and DC twins 1, whereas De Paepe et al. reported a lower placental weight and fetal:placental weight ratio in MC compared to DC twins 3.

Intrauterine growth restriction (IUGR) occurs in approximately 12-15% of twin pregnancies, with a similar incidence in DC and MC twins 7. We identified only one paper that studied placental weight in MC twins with IUGR 8. The authors found a significantly higher birth weight to placental weight ratio (BW/PW) in an IUGR MC twin compared to the appropriate for gestational age co-twin (6.4 *vs* 4.0, respectively, *p*<0.001).

Variations in the process of placentation in twin pregnancies influence nutrient supply to one or both fetuses. It is necessary to identify certain placental characteristics and behaviors in some fetal conditions to understand the contribution of the placenta in determining disorders later in life 9. Therefore, in the present study, we aimed to compare the placental weight and BW/PW ratio of IUGR and non-IUGR (nIUGR) MC and DC twins. In addition, we describe the frequency and association of cord insertion type and placental lesions with placental weight and the BW/PW ratio in these twins.

## MATERIALS AND METHODS

This was a retrospective analysis of placentas from twin pregnancies delivered at the Department of Obstetrics and Gynecology, São Paulo University Medical School Hospital, between July 2006 and December 2014.

A database search was performed to identify all twin pregnancies with a gestational age at delivery of 24 or more weeks; both twins alive at birth; and the absence of structural or chromosomal abnormalities in the newborns, twin-to-twin transfusion syndrome, and placental complications such as placenta previa, placenta accreta or placental abruption. All twin placentas delivered in our hospital are routinely sent to the Department of Pathology for gross and histopathologic examination; the information collected includes weight, type of umbilical cord insertion and twists, number of umbilical arteries, type of membrane insertion, placental chorionicity and amnionicity. All placentas received at the pathology laboratory had already been fixed in formaldehyde for 24 hours. The cord and membranes were trimmed before weighing. DC fused placentas and MC placentas were not separated for weighing; therefore, only the total placental weight was recorded for all cases (the sum of DC separated, DC fused and MC placentas). The type of cord insertion was defined as follows: paracentral; marginal, insertion at the edge of the placental disc; and velamentous, insertion into the fetal membranes. For the purpose of the present analysis, we considered paracentral and peripheral (marginal + velamentous) insertion types. The following placental lesions were assessed: chorangiosis, intraplacental hematoma, villous immaturity, villous infarction, and chronic villitis. The histological diagnosis of these lesions was based on previously established criteria 10.

Gestational age was calculated from the first day of the last menstrual period (LMP) and confirmed by ultrasound crown-rump length measurement during the first trimester or by an estimate based on multiple ultrasound parameters (biparietal, head circumference, abdominal circumference and femur length) of the largest fetus during the second trimester. When the LMP was uncertain or unknown, or there was discrepancy between gestational age based on LMP and ultrasound, gestational age was determined based on the earliest ultrasound findings. Growth restriction was considered when the birth weight was below the 10^th^ percentile according to the growth chart for twins 11. Either one or both twins could be growth restricted. The BW/PW ratio was calculated by dividing the total birth weight (the sum of both twins’ birth weight) by the placenta weight.

The primary end-points were a comparison of the placental weight and BW/PW ratio in IUGR and nIUGR twins according to chorionicity and a description of the frequency and association of cord insertion type and placental lesions with placental weight and BW/PW ratio in IUGR and nIUGR twins according to chorionicity.

### Statistical analysis

Numerical data are presented as the mean and standard deviation or the median, minimum and maximum when appropriate. Categorical data are presented as relative and absolute frequencies. The associations between qualitative categorical variables were investigated using Chi-square or Fisher’s exact tests, when appropriate. For quantitative variables, the groups were compared with the Mann-Whitney U test or Student’s t test, depending on the data characteristics. Pearson’s correlation coefficient (r) was utilized to measure the linear relationship between two quantitative variables.

A *p*-value<0.05 was considered to indicate statistical significance. The data were analyzed using Statistical Package for the Social Sciences (SPSS version 20, IBM SPSS Incorporated, Chicago, IL, USA).

### Ethics

The study protocol was registered and approved by the Institutional Ethics Review Board (CAPPesq 1.412.280).

## RESULTS

The database search identified 468 twin pregnancies with delivery at 24 or more weeks gestation in our hospital. One hundred forty-four of these pregnancies were excluded for the following reasons: fetal malformation or chromosomal abnormalities affecting one or both fetuses (n= 49), fetal death of one or both twins (n=28), twin-to-twin transfusion syndrome (n=22), no placental pathological examination (n=21), no placental weight (n=16), congenital infection (n=1), placenta accreta (n=5), placenta previa (n=1) and placental abruption (n=1). The final analysis was based on 324 twin pregnancies, of which 32.4% (n=105) were MC, and 67.6% (n=219) were DC.

Maternal characteristics and pregnancy information according to chorionicity are summarized in Table 1. Women pregnant with DC twins were significantly older and more likely to be multiparous compared to women pregnant with MC twins. No significant differences were observed for other maternal characteristics (Table 1). MC pregnancies had a younger gestational age at delivery, lower total birth weight, lower total placental weight and higher mean birth weight discordance than their DC counterparts. No difference was observed in the number of placental lesions between MC and DC placentas (Table 1).

**Figure 1:**
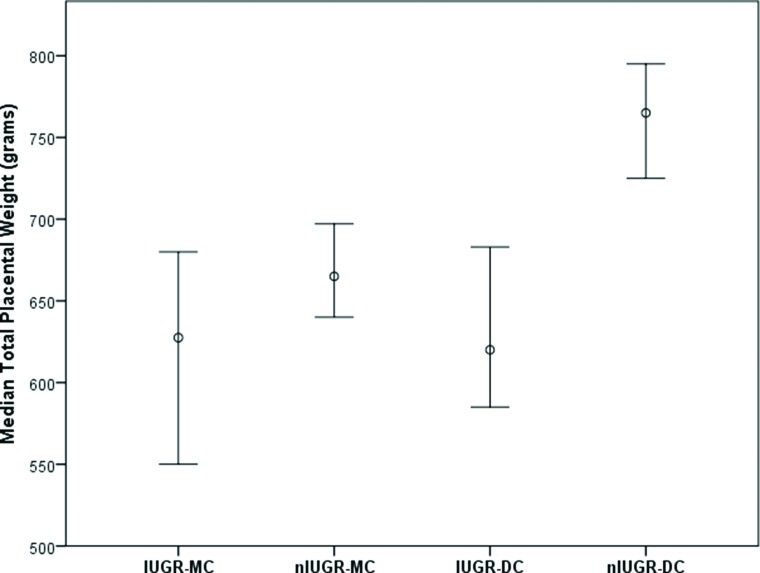
Median total placental weight and 95% confidence intervals for intrauterine growth restriction (IUGR) and non-IUGR (nIUGR) cases according to chorionicity (MC, monochorionic; DC, dichorionic).

Table 2 presents the placental weight in IUGR and nIUGR MC and DC twins as a function of gestational age at delivery. A significantly lower mean placental weight was observed for IUGR compared to nIUGR twins considering those at all gestational ages at birth regardless of chorionicity (Figure 1), those at 32 to 34 weeks gestation for the MC group and those between 29 and 31 weeks or at or above 35 weeks for the DC group (Table 2). The BW/PW ratio was significantly lower in MC IUGR twins compared to nIUGR twins (*p*=0.006) with a gestational age at birth of at least 35 weeks (Table 3). However, no differences were observed overall or for other gestational age periods for either MC or DC twins. Both twins were diagnosed with IUGR in only 12 cases, seven sets in the MC group (17.5%, 7/40) and five sets in the DC group (8.77%, 5/57). There was no difference in the BW/PW ratio between the IUGR and nIUGR groups when either one or both MC or DC twins were affected (Table 3).

No correlation was observed between maternal stature and placental weight (r=0.119, r=0.035 and r=0.132) or BW/PW ratio (r=0.075, r=0.064 and r=0.077) for all included pregnancies or according to chorionicity (MC and DC), respectively. Additionally, no difference was observed between multiparous and non-multiparous women in terms of placental weight (*p*=0.39, *p*=0.38 and *p*=0.91) or BW/PW ratio (*p*=0.41, *p*=0.89 and *p*=0.53) for all included pregnancies or according to chorionicity (MC and DC), respectively.

In 283 (87.34%) cases, we had information from both twins on the type of umbilical cord insertion at the placenta. Paracentral umbilical cord insertion was observed for both twins in 47.3% of cases (n=134), and peripheral insertion (marginal or velamentous) was observed in at least one twin in 52.7% of cases (n=149). Peripheral umbilical cord insertion in at least one twin was more frequent in MC (n=74; 78.7%) compared to DC cases (n=75; 39.7%), whereas paracentral insertion was more frequent in DC (n=114; 60.3%) compared to MC cases (n=20; 21.3%). In MC twins, peripheral cord insertion was significantly associated with IUGR (IUGR=91.7% *vs* nIUGR=70.7%; *p*=0.02). No association was observed between cord insertion type (paracentral *vs* peripheral) and IUGR (65.4% and 34.6%) or nIUGR (58.4% and 41.6%) in DC twins (*p*=0.41). Regarding paracentral or peripheral insertion, no difference was observed in mean placental weight for MC (677.38 g±136.50 g and 648.28 g±129.43 g; *p*=0.38) or DC twins (746.42 g±172.31 g and 705.04 g±198.93 g; *p*=0.13) or in the BW/PW ratio for MC (6.02±1.05 and 6.09±1.20; *p*=0.82) or DC twins (6.38±1.11 and 6.28±1.26; *p*=0.56). When cord insertion type was analyzed in the IUGR and nIUGR groups as a function of chorionicity (Table 4), the only significant difference was observed for DC twins, with a lower placental weight in IUGR twins with peripheral cord insertion compared to IUGR twins with paracentral cord insertion (*p*=0.02; Table 4).

Six types of placental histopathological lesions were analyzed (Table 5). No association was observed between chorionicity and the presence or absence of a placental lesion (*p*=0.99). Chorangiosis was significantly more frequent in MC placentas compared to DC placentas (*p*=0.03), but no differences were observed for the other types of placental lesions (Table 5). In addition, we did not find any significant differences in the frequency of placental lesions between the IUGR and nIUGR groups of MC or DC twins (Table 5). However, intraplacental hematoma was more frequent among IUGR DC twins compared to nIUGR DC twins. There was no significant association between the presence of a placental lesion and placental weight in any of the included groups (Table 6).

## DISCUSSION

The present study demonstrated that overall placental weight is significantly lower in MC and DC IUGR twins compared to nIUGR twins, but there was no difference in the overall BW/PW ratio. Regarding gestational age at birth and placental weight in IUGR and nIUGR twins, the results were not the same for MC and DC twins (Table 2). For births at 32 to 34 weeks, IUGR MC twins had a significantly lower placental weight compared to their nIUGR counterparts, and these twins were delivered earlier due to the low placental reserve for fetal nutrition. Interestingly, the BW/PW ratio at this period was higher, but not significantly different, in IUGR twins compared to nIUGR twins, indicating that both the placenta and the fetus are small in IUGR twins. At 35 weeks and older, no difference between IUGR and nIUGR in MC twins was observed in placental weight, but there was a significantly lower BW/PW ratio; this is perhaps because the placenta in these IUGR cases is bigger with greater functionality, and these are probably less severe cases in which the pregnancy can be maintained until a later gestational age. Furthermore, MC twins with IUGR that require early delivery have a very small placenta resulting from unequal placenta sharing and unbalanced vascular anastomoses 12,13, with consequent severe growth restriction; conversely, the cases with later delivery have an equally shared placenta, and the growth restriction may be more related to unbalanced inter-twin transfusion mediated by vascular anastomoses in the placenta rather than placenta size 12,13.

In a previous study involving MC twin placentas, the authors obtained the individual weights of the placentas by transecting the placenta at the vascular equator and weighing each individual disc separately 8. Similar to our study, the authors observed a significantly lower placental weight for the IUGR twin but a higher BW/PW ratio compared to that of the adequate birthweight co-twin; these differences were not observed in twin pairs without IUGR 8. In our study, we did not separate the placentas prior to weighing; however, the total weight (sum of placental weight) for either MC or DC twins was significantly lower in IUGR cases compared to nIUGR cases, in agreement with the results from Chang et al. 8. In contrast to their results, we did not observe an increased BW/PW ratio, which may be due to the combined placental weight and the possible influence of the co-twin’s placental weight. Nevertheless, the increased BW/PW ratio in their study was due to the very low placental weight of the IUGR twin, which supports the assumption that these cases of early, severe IUGR originate from unequal placental sharing 12,13. Few studies have investigated placental weight and the BW/PW ratio in MC IUGR twins, which limits further conclusions. We do not believe that weighing the MC placentas separately would have yielded a better understanding because the placental volume and size cannot be examined separately in antenatal practice. Furthermore, we agree with the observation by De Paepe et al. that the weight of separate placentas in MC cases inevitably remains an estimate, as the superficial choriovascular distribution may not necessarily correlate with functional parenchymal partition 3.

For DC twins, the placental weight was significantly lower in IUGR twins compared to nIUGR twins at gestational ages at birth of 29 to 31 weeks and at 35 weeks and above, and no differences in the BW/PW ratio were observed in these periods. Nevertheless, the BW/PW ratio could have been influenced by the birth weight and placental weight of the appropriate for gestation age co-twin. Further studies examining separate placentas in DC IUGR and nIUGR twins may elucidate this matter. Due to the independent placental circulation, we allow DC twin pregnancies with one IUGR twin to be delivered at a later gestational age, which benefits the larger twin, as fetal well-being can be controlled in a predictable way. This explains the significantly lower placental weight after 35 weeks gestation in the IUGR group.

We observed differences in the cord insertion type for both MC and DC placentas. In accordance with a previous study 3, we found a higher frequency of peripheral cord insertion in MC gestations. In spite of the association between peripheral cord insertion and IUGR MC, there was no relation between cord insertion type and placental weight or BW/PW ratio (Table 4). This finding suggests that the influence of peripheral cord insertion on birth weight is more related to unequal vascularization 12,13 in these twins rather than placenta size.

In contrast, in DC twins, the cord insertion type did not influence the placental weight or BW/PW ratio in either IUGR or nIUGR twins (Table 4), which may be explained by other mechanisms of fetal growth restriction, such as placental functional impairment, suboptimal implantation sites or other environmental factors 6. De Paepe et al. also did not find a significant correlation between cord insertion type and fetal/placental ratio in DC twins 3.

Histopathological examination of the placenta can identify specific lesions that, in theory, influence fetal growth abnormalities 13,14. Our analysis revealed a similar number of placental lesions in MC and DC twins, with no difference between IUGR and nIUGR cases independent of chorionicity (Tables 5 and 6). Regarding specific placental lesions, intraplacental hematoma was more frequent in DC IUGR twins, and villitis was more frequent in MC IUGR cases compared to their nIUGR counterparts; however, there were no significant differences for any of the lesions. We observed an association between lower placental weight and the number of placental lesions in DC IUGR twins, although this association did not reach significance. However, in DC twins, Eberly et al. observed that the lighter twin’s placenta had more lesions (77.8%) than that of the heavier twin (34.6%) 6. Based on our findings, we cannot assume that IUGR in either MC or DC twins is due to the type or presence of placental lesions, despite the possible contribution of these lesions to IUGR in DC twins.

This study involved a retrospective analysis, but very few studies have examined placental weight and the BW/PW ratio in IUGR and nIUGR twins according to chorionicity. The main limitation of the study was the lack of data on placental vascularization in MC pregnancies, which could provide valuable knowledge for understanding the relation between birth weight and placental weight. Another limitation was that the weight of DC placentas was considered as the sum of the weight of both placentas. Indeed, we could have included only non-fused placentas and presented the separate placental weight; however, we preferred to unify the technical analysis with that of MC placentas. Notwithstanding these limitations, this study provides insights regarding placental involvement in IUGR in MC and DC twins.

In conclusion, for both MC and DC twins, the placental weight is lower in IUGR cases than in non-IUGR cases, while the overall BW/PW ratio is similar in IUGR and non-IUGR twins. The mechanism by which low placental weight influences the BW/PW ratio in IUGR MC and DC twins needs to be determined in larger prospective studies that include antenatal Doppler flow examinations, separate and combined placental weights, vascularization studies and histopathology examinations.

## AUTHOR CONTRIBUTIONS

Souza MA developed the project, collected the data and wrote the manuscript. Brizot ML developed the project, participated in the data analysis and wrote the manuscript. Biancolin SE developed the project, participated in the data analysis and wrote the manuscript. Schultz R performed the placental pathology examinations and participated in the data collection and analysis. de Carvalho MH participated in the manuscript writing, data analysis, and manuscript revision. Francisco RP revised the manuscript and contributed to relevant discussions. Zugaib M revised the manuscript.

## Figures and Tables

**Table 1:** Baseline characteristics of study population according to chorionicity.

	Monochorionic n=105	Dichorionic n=219	*p*
**Maternal characteristics**			
Age (yr), mean ± SD	26.9 (6.68)	29.58 (6.45)	0.01*
Stature (m), mean ± SD	1.60 (0.06)	1.61 (0.06)	0.37*
White ethnicity, n (%)	61 (58.1)	115 (52.5)	0.40#
Multiparous, n (%)	19 (18.1)	72 (32.9)	0.006#
Smoking during pregnancy, n (%)	11 (10.5)	41 (18.7)	0.05#
Alcohol abuse during pregnancy, n (%)	0 (0)	5 (2.3)	0.18§
Illicit substance use during pregnancy, n (%)	1 (1)	8 (3.7)	0.28§
Medical disorders prior to pregnancy, n (%)**	28 (26.7)	47 (21.6)	0.33#
Gestational complications	20 (19)	59 (26.9)	0.13#
**Perinatal characteristics**			
Twin growth restriction, n (%)	40 (38.1)	57 (26)	0.04#
Birth weight discordance (%), mean ± SD	15.63 (13.51)	12.3 (11.05)	0.03*
Gestational age at delivery, mean ± SD	34.45 (2.55)	35.55 (2.89)	0.001*
Total birth weight (g), mean ± SD	3913.98 (919.17)	4537.36 (1106.89)	<0.001*
**Placental characteristics**			
Total placental weight (g), mean ± SD	645.94 (133.02)	730.33 (186.39)	<0.001*
Birth weight/placental weight ratio	6.12 (1.16)	6.32 (1.18)	0.17
Number of lesions, mean ± SD	0.60 (0.83)	0.57 (0.75)	0.79*

SD, standard deviation; yr, years.

* Student’s t test;

# Chi-square test;

§ Fisher’s exact test

** Medical disorders prior to pregnancy: chronic hypertension, diabetes, asthma, cardiopathy, hypothyroidism, thrombophilia, or chronic kidney disease.

**Table 2:** Total placental weight in intrauterine growth restriction (IUGR) and non-IUGR (nIUGR) monochorionic and dichorionic twins as a function of gestational age (GA) at birth.

	Median total birth weight in grams (range)				Median total placental weight in grams (range) (number of cases)					
GA at Birth (wks)	Monochorionic, N=105		Dichorionic, N=219		Monochorionic, N=105			Dichorionic, N=219		
	IUGR (n=40)	nIUGR (n=65)	IUGR (n=57)	nIUGR (n=162)	IUGR (n=40)	nIUGR (n=65)	*p*	IUGR (n=57)	nIUGR (n=162)	*p*
**≤28**	-	1965 (1850-3053)	-	1620 (1280-2440)	(0)	415 (330-575) (6)	-	(0)	395 (185-617) (8)	-
**29–31**	2390 (2220-2560)	2936 (2370-3394)	2040 (1770-2360)	2810 (2370-3600)	530 (520-540) (2)	529 (465-730) (7)	0.99*	335 (230-500) (3)	542 (365-710) (14)	0.03*
**32–34**	3185 (1970-3920)	3845 (2950-4820)	3320 (2640-4120)	3980 (3090-5280)	494.35 (340-690) (16)	650 (540-820) (16)	<0.001*	603 (420-960) (13)	684 (475-1035) (22)	0.20*
**≥35**	3990 (3010-4670)	4819 (3840-5950)	4350 (3260-5200)	5280 (3880-6570)	692.5 (505-830) (22)	696 (450-990) (36)	0.25*	645 (450-1130) (41)	807.5 (505-1340) (118)	<0.001*
**All (24-38)**	3650 (1970-4670)	4400 (1850-5950)	4180 (1770-5200)	5035 (1280-6570)	627.5 (340-830) (40)	665 (330-990) (65)	0.02**	620 (230-1130) (57)	765 (185-1340) (162)	<0.001**

Wks, weeks;

* Mann-Whitney U test;

** Student’s t test.

**Table 3:** Birth weight/placental weight ratio (BW/PW) in intrauterine growth restriction (IUGR) and non-IUGR (nIUGR) monochorionic and dichorionic twins as a function of gestational age (GA) at birth.

BW/PW, median (range) (number of cases)						
GA at Birth (weeks)	Monochorionic, N=105		*p*	Dichorionic, N=219		*p*
	IUGR (n=40)	nIUGR (n=65)		IUGR (n=57)	nIUGR (n=162)	
**≤28**	(0)	5.12 (3.28-7.53) (6)	-	(0)	4.11 (3.51-6.91) (8)	-
**29–31**	4.50 (4.26-4.74) (2)	5.55 (3.20-6.40) (7)	0.33*	5.28 (4.72-8.86) (3)	5.01 (4.12-7.12) (14)	0.51*
**32–34**	6.21 (3.53-8.76) (16)	5.85 (4.60-7.04) (16)	0.32*	5.28 (3.73-7.92) (13)	6.08 (4.16-9.22) (22)	0.18*
**≥35**	5.92 (4.12-8.02) (22)	6.83 (5.12-9.93) (36)	0.006*	6.59 (4.60-9.29) (41)	6.49 (3.99-9.23) (118)	0.67*
**All (24-38)**	5.93 (3.53-8.76) (40)	6.20 (3.24-9.93) (65)	0.36**	6.38 (3.73-9.29) (57)	6.32 (3.51-9.23) (162)	0.68**
**One twin with IUGR (24-38)**	5.94 (3.53- 8.77) (33)	6.20 (3.24-9.93) (65)	0.454*	6.38 (3.74-9.30) (52)	6.32 (3.51-9.23) (162)	0.662*
**Both twins with IUGR (24-38)**	5.27 (4.28- 7.36) (7)	6.20 (3.24-9.93) (65)	0.242*	5.77 (5.25- 7.13) (5)	6.32 (3.51-9.23) (162)	0.659*

* Mann-Whitney U test;

** Student’s t test.

**Table 4:** Placental weight and birth weight to placental weight ratio (BW/PW) in intrauterine growth restriction (IUGR) and non-IUGR (nIUGR) monochorionic and dichorionic twins as a function of cord insertion type.

	Monochorionic						Dichorionic					
	IUGR		*p* *	nIUGR		*p* *	IUGR		*p* *	nIUGR		*p* *
	Paracentral N=3	Peripheral N=33		Paracentral N=17	Peripheral N=41		Paracentral N=34	Peripheral N=18		Paracentral N=80	Peripheral N=57	
**Placental weight**	680 (530-730)	630 (390-830)	0.66	650 (417-990)	675 (330-950)	0.80	684 (230-1130)	582.5 (335-895)	0.02	747.5 (365-1340)	780 (185-1095)	0.71
**BW/PW ratio**	5.89 (4.12-6.73)	5.94 (3.53-8.76)	0.66	5.80 (4.60-8.11)	6.09 (3.24-9.93)	0.58	6.43 (4.38-8.96	6.06 (4.14-9.29)	0.55	6.39 (3.79-9.23)	6.26 (3.51-9.22)	0.53

* Mann-Whitney U test.

**Table 5:** Comparison of placental lesions according to chorionicity and intrauterine growth restriction (IUGR) or non-IUGR (nIUGR).

Placental Lesions	Total N (%)	Chorionicity		*p*	Monochorionic		Dichorionic			*p*
		MC N=105	DC N=219		IUGR N=40	nIUGR n=65	*p*	IUGR N=57	nIUGR n=162	
**At least one lesion**	113 (34.9)	41 (39)	72 (32.9)	0.32*	20 (50)	21 (32.3)	0.09*	21 (36.8)	51 (31.5)	0.51*
**Chorangiosis**	34 (10.5)	17 (16.2)	17 (7.8)	0.03*	8 (20)	9 (13.8)	0.42*	2 (3.5)	15 (9.3)	0.25#
**Fibrin deposition**	16 (4.9)	3 (2.9)	13 (5.9)	0.28#	2 (5)	1 (1.5)	0.55#	2 (3.5)	11 (6.8)	0.52#
**Intraplacental hematoma**	35 (10.8)	10 (9.5)	25 (11.4)	0.70*	4 (10)	6 (9.2)	0.99#	11 (19.3)	14 (8.6)	0.05*
**Villous immaturity**	13 (4)	7 (6.7)	6 (2.7)	0.12#	4 (10)	3 (4.6)	0.42#	1 (1.8)	5 (3.1)	0.99#
**Villous infarction**	40 (12.3)	13 (12.4)	27 (12.3)	0.99*	4 (10)	9 (13.8)	0.76#	11 (19.3)	16 (9.9)	0.09*
**Chronic villitis**	7 (2.2)	3 (2.9)	4 (1.8)	0.68#	3 (7.5)	0 (0)	0.05#	0 (0)	4 (2.5)	0.57#

MC, Monochorionic; DC, Dichorionic;

* Chi-square test;

# Fisher’s exact test.

**Table 6:** Total placental weight according to the presence or absence of placental lesions in monochorionic and dichorionic intrauterine growth restriction (IUGR) and non-IUGR (nIUGR) twins.

Groups	No placental lesions Mean (±SD)	At least one lesion Mean (±SD)	*p* *
**All placentas, n=324**	708.99 (182.87)	691.75 (160.26)	0.39
**Monochorionic**			
** All, n=105**	647.88 (136.91)	642.91 (128.32)	0.85
** IUGR, n=40**	607.19 (137.80)	609.55 (117.17)	0.95
** nIUGR, n=65**	666.38 (133.99)	674.68 (133.12)	0.81
**Dichorionic**			
** All, n=219**	735.60 (194.06)	719.56 (170.46)	0.55
** IUGR, n=57**	662.16 (155.12)	612.79 (158.55)	0.25
** nIUGR, n=162**	759.42 (199.98)	763.52 (156.36)	0.89

* Student’s t test.
